# Prognostic value of total lesion glycolysis of baseline ^18^F-fluorodeoxyglucose positron emission tomography/computed tomography in diffuse large B-cell lymphoma

**DOI:** 10.18632/oncotarget.13180

**Published:** 2016-11-07

**Authors:** Mingge Zhou, Yumei Chen, Honghui Huang, Xiang Zhou, Jianjun Liu, Gang Huang

**Affiliations:** ^1^ Department of Nuclear Medicine, Ren Ji Hospital, School of Medicine, Shanghai Jiao Tong University, Shanghai, China; ^2^ Department of Hematology, Ren Ji Hospital, School of Medicine, Shanghai Jiao Tong University, Shanghai, China

**Keywords:** DLBCL, prognosis, PET/CT, TLG, MTV

## Abstract

**Purpose:**

We evaluated the prognostic value of total lesion glycolysis (TLG) measured in baseline ^18^F-fluorodeoxyglucose positron emission tomography/computed tomography (^18^F-FDG PET/CT) in diffuse large B-cell lymphoma (DLBCL) treated with rituximab plus cyclophosphamide, doxorubicin, vincristine, and prednisone (R-CHOP).

**Methods:**

A total of 91 patients with newly diagnosed DLBCL underwent ^18^F-FDG PET/CT scans before R-CHOP therapy. Metabolic tumor volume (MTV) was measured with the marginal threshold of normal liver mean standard uptake value (SUVmean) plus 3 standard deviations (SD). TLG was the sum of the products of MTV and SUVmean in all measured lesions. The predictive value was estimated by Log-rank test and Cox-regression analysis.

**Results:**

Median follow-up was 30 months (range, 5-124 months). The 5-year estimated progression-free survival (PFS) of the low and high TLG group were 83% and 34%, respectively (p<0.001). The 5-year overall survival (OS) of the same groups were 92% and 67%, respectively (p<0.001). Patients with high TLG level were more likely to relapse than those with low TLG level even though they had got complete or partial remission in R-CHOP therapy (40% versus 9%, p=0.012). Multivariate analysis revealed TLG was the only independent predictor for PFS (Hazard ratio=5.211, 95% confidence interval=2.210-12.288, p<0.001) and OS (Hazard ratio=9.136, 95% confidence interval=1.829-45.644, p=0.002). Other factors including MTV, National Comprehensive Cancer Network International Prognostic Index (NCCN-IPI) and Ann Arbor Stage were not independently predictive for survivals.

**Conclusion:**

Baseline TLG is the only independent predictor for PFS and OS in DLBCL patients treated with R-CHOP therapy.

## INTRODUCTION

Diffuse large B-cell lymphoma is the most common form of non-Hodgkin's lymphoma, accounting for one-third of all adult lymphoma. During the last decade, R-CHOP therapy has markedly improved patients' outcomes [1]. However, approximately one–third of the patients will develop relapsed or refractory disease that mainly results in morbidity and mortality [2]. So, it is crucial to identify those who are likely to have poor outcomes [3]. IPI has been used for predicting the prognosis in patients with aggressive non-Hodgkin's lymphoma for more than 20 years, but the introduction of rituximab weakens its' discriminating power [4, 5]. NCCN-IPI also provides some information of risk stratification [6], but is still not enough for clinicians. More prognostic factors should be explored.

^18^F-fluorodeoxyglucose positron emission tomography/computed tomography is a powerful tool of showing the fusion of anatomical structure and metabolism of lesions. It is now widely used for the management of DLBCL [7–9]. The association between SUV and prognosis of DLBCL has been widely studied. However, not many studies are available to evaluate the prognostic value of MTV and TLG in DLBCL and these studies draw different conclusions [10–21].

The purpose of the present study is to demonstrate the prognostic value of TLG derived from baseline PET/CT, and to compare TLG with other clinical factors, in newly diagnosed DLBCL patients treated with R-CHOP therapy.

## RESULTS

### Patient characteristics

Baseline demographic, clinical and pathologic characteristics of 91 patients were summarized in Table 1. The median age was 56 years old (range, 17-83 years old), and the male to female ratio was 0.93:1. Complete remission (CR) and partial remission (PR) were achieved in 79 out of 91 (87%) patients after 6 or 8 cycles of R-CHOP therapy. After a median follow-up of 30 months (range, 5-124 months), 27 patients had disease relapse or progression and 11 patients died. The 5-year PFS and 5-year OS were estimated in life tables, shown as 65% and 82%, respectively.

**Table 1:** Characteristics of DLBCL patients

Patient characteristics (n=91)	No. of patients (%)
Age	56±14
≤60 y	53(58)
>60 y	38(42)
Female Sex	47(52)
NCCN-IPI score	
low and low-intermediate group (0-3 scores)	52(57)
high-intermediate and high group (4-8scores)	39(43)
Performance status	
ECOG 0-1	71(78)
ECOG >1	20(22)
B symptoms at presentation	50(55)
Ann Arbor Stage	
I/II	34(37)
III/IV	57(63)
Extranodal sites >1	36(40)
Elevated LDH level	36(40)

Abbreviations: DLBCL, diffuse large B-cell lymphoma; NCCN-IPI, National Comprehensive Cancer Network-International Prognostic Index; ECOG, Eastern Cooperative Oncology Group; LDH, lactate dehydrogenase.

### Clinical characteristics of patients in relation to MTV and TLG

Table 2 revealed the difference in clinical characteristics between the dichotomized MTV and TLG groups. Pearson's chi-square test shows NCCN-IPI score, Ann Arbor stage, B symptoms, performance status and LDH level are significantly associated with MTV and TLG. Those patients with high MTV and TLG levels usually possessed the following characteristics: high NCCN-IPI scores, stage III/IV, B symptoms, poor performance status or elevated LDH levels.

**Table 2:** Comparison between low and high MTV/TLG groups

Clinical Factors	No. of patients (%)			P value ^b^	No. of patients (%)		
	Total	Low MTV ^a^	High MTV ^a^		Low TLG ^a^	High TLG ^a^	P value ^b^
Age				0.607			0.737
≤60 y	53	28(61)	25(56)		26(57)	27(60)	
>60 y	28	18(39)	20(44)		20(43)	18(40)	
NCCN-IPI score				<0.001			<0.001
0-3	52	37(80)	15(33)		35(76)	17(38)	
4-8	39	9(20)	30(67)		11(24)	28(62)	
Ann Arbor Stage				0.003			0.037
I/II	34	24(52)	10(22)		22(48)	12(27)	
III/IV	57	22(48)	35(48)		24(52)	33(73)	
B symptoms				<0.001			0.008
No	41	29(63)	12(27)		27(59)	14(31)	
Yes	50	17(37)	33(73)		19(41)	31(69)	
Performance status				0.002			0.037
ECOG 0-1	71	42(91)	29(64)		40(87)	31(69)	
ECOG >1	20	4(9)	16(36)		6(13)	14(31)	
No. of extranodal sites				0.072			0.17
0-1	55	32(70)	23(51)		31(67)	24(53)	
2 or more	36	14(30)	22(49)		15(33)	21(47)	
LDH level				0.002			0.002
normal	55	35(85)	20(44)		35(76)	20(44)	
elevated	36	11(24)	25(56)		11(24)	25(56)	

Abbreviations: MTV, metabolic tumor volume; TLG, total lesion glycolysis; NCCN-IPI, National Comprehensive Cancer Network-International Prognostic Index; ECOG, Eastern Cooperative Oncology Group; LDH, lactate dehydrogenase.

a MTV and TLG were dichotomized by respective median values.

b Pearson's chi-square test.

### Survival analysis and prediction of survivals

**Figure 1:**
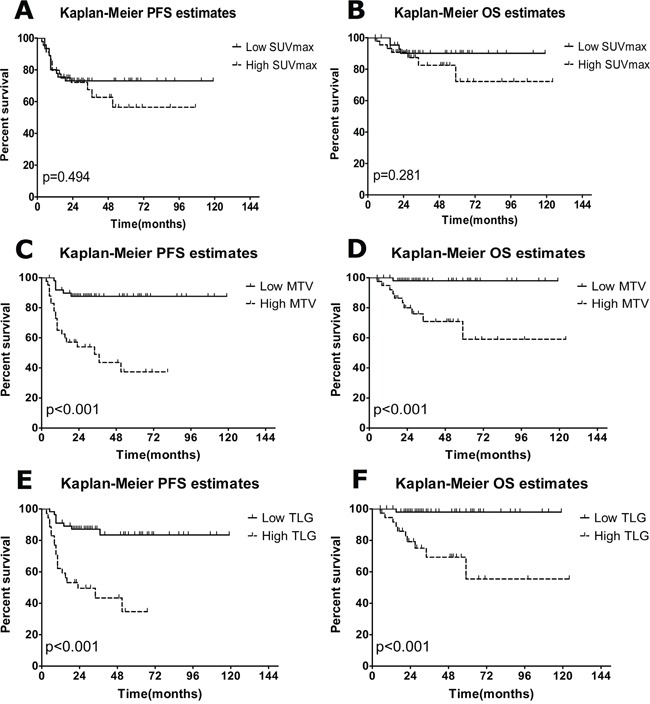
Estimates of PFS and OS according to parameters in baseline PET/CT **A, C, E.** Kaplan-Meier estimates of PFS; **B, D., E** Kaplan-Meier estimates of OS. High, above the cutoff values from ROC analysis; low, at or below the cutoff values from ROC analysis.

**Figure 2:**
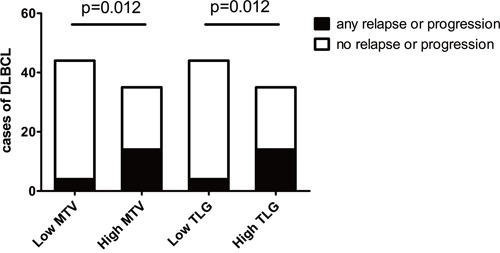
Relapse or progression in patients achieving remission in R-CHOP therapy High, above the median values; low, at or below the median values.

**Figure 3:**
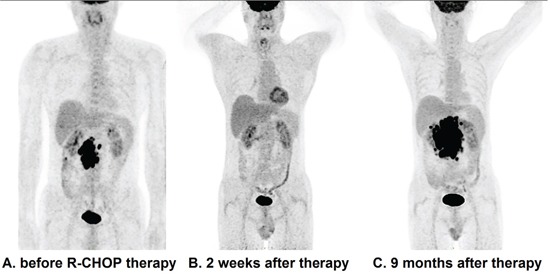
^18^F-FDG PET images of a recurrent case The baseline PET image shows high tumor burden with TLG of 1244g **A.** Although no hyper-metabolic lesions were seen on PET images after 6 cycles of R-CHOP therapy **B.**, the patient experienced relapse nine months after the R-CHOP therapy **C.**

The descriptions of baseline PET metabolic parameters including SUVmax, MTV and TLG are summarized in Table 3. High MTV and TLG levels were significantly associated with poor PFS and OS, according to Kaplan-Meier curves and Log-rank test (Figure 1). The 5-year PFS of the low and high TLG group were 83% and 34%, respectively (p<0.001). The 5-year OS of the same groups were 92% and 67%, respectively (p<0.001). Other factors including MTV, NCCN-IPI, Ann Arbor stage, B symptoms and LDH level were also associated with PFS and OS, according to the results of univariate analysis shown in Table 4. SUVmax failed to discriminate patients with poor PFS or OS (p=0.494, p=0.282, respectively). Interestingly, we found the patients with higher MTV or TLG level could have more risk to suffer from disease relapse or progression, even if they had achieved remission in R-CHOP therapy. Figure 2 shows, in 79 patients who got remission in R-CHOP therapy, 14 out of 35 (40%) high-TLG patients have experienced disease relapse or progression, while only 4 out of 44 (9%) low-TLG patients have experienced relapse or progression (χ^2^=6.323, p=0.012). It is the same in the analysis of MTV, showing 14 out of 35 (40%) versus 4 out of 44 (9%) (χ^2^=6.323, p=0.012). Figure 3 shows an example of disease relapse after getting CR in R-CHOP therapy. The baseline PET image before therapy showed high tumor burden with TLG of 1244g (Figure 3A). After 6 cycles of R-CHOP therapy, no hyper-metabolic lesions were seen on the PET image (Figure 3B). But the patient experienced relapse nine months after the R-CHOP therapy (Figure 3C).

**Table 3:** Baseline PET/CT parameters

Parameter	Median (interquartile range)	ROC curve for PFS					ROC curve for OS				
		AUC (95%CI)	P value	Cutoff	Sensitivity	Specificity	AUC (95%CI)	P value	Cutoff	Sensitivity	Specificity
SUVmax	19.3(11.7-28.8)	0.527(0.402-0.653)	0.68	19.0	63%	52%	0.604(0.467-0.741)	0.265	15.8	91%	44%
MTV (cm^3^)	50.7(17.4-150.9)	0.720(0.599-0.840)	0.001	70.0	78%	69%	0.813(0.713-0.913)	0.001	78.0	91%	64%
TLG(g)	497.3(104.0-1451.6)	0.714(0.591-0.836)	0.001	826.5	70%	75%	0.81(0.706-0.915)	0.001	726.0	91%		66%

Abbreviations: SUVmax, maximum standard uptake value; ROC curve, receiver-operating characteristic curve; PFS, progression-free survival; OS, overall survival; AUC, area under curve; CI, confidence interval.

**Table 4:** Univariate analysis for survivals

Parameters	5-year PFS	P value ^b^	5-year OS	P value ^b^
SUVmax ^a^		0.494		0.282
Low	73%		90%	
High	57%		72%	
MTV ^a^		<0.001		<0.001
Low	88%		98%	
High	37%		60%	
TLG ^a^		<0.001		<0.001
Low	83%		92%	
High	34%		67%	
NCCN-IPI score		0.039		0.036
0-3	76%		92%	
4-8	51%		71%	
Ann Arbor stage		0.02		0.046
I/II	82%		94%	
III/IV	56%		76%	
B symptoms		<0.001		0.027
No	86%		93%	
Yes	46%		70%	
LDH level		0.013		0.015
normal	77%		88%	
elevated	48%		73%	
Subtype		0.761		0.762
GCB	74%		88%	
non-GCB	65%		80%	

Abbreviations: PFS, progression-free survival; OS, overall survival; SUVmax, maximum standard uptake value; MTV, metabolic tumor volume; TLG, total lesion glycolysis; NCCN-IPI, National Comprehensive Cancer Network-International Prognostic Index; LDH, lactate dehydrogenase; GCB, germinal centre B-cell.

a SUVmax, MTV and TLG were dichotomized by cutoff values from ROC analysis.

b Log-rank test.

The results of multivariate analysis showed TLG was the only independent predictor of PFS and OS (HR=5.211, 95%CI=2.210-12.288, p< 0.001; HR=9.136, 95%CI=1.829-45.644, p=0.002, respectively). Ann Arbor stage trended to be an independent predictor of PFS and OS (p=0.094, p=0.069, respectively). MTV, NCCN-IPI, B symptoms, LDH and Ki-67 failed to be independently predictive. All the results of Cox-regression were summarized in Table 5.

**Table 5:** Multivariate analysis for survivals

Parameters	analysis for PFS ^b^			analysis for OS ^b^		
	HR	95%CI	P value	HR	95%CI	P value
MTV ^a^	–	–	0.151	–	–	0.103
TLG ^a^	5.211	2.210-12.288	<0.001	9.136	1.829-45.644	0.002
NCCN-IPI	–	–	0.433	–	–	0.083
Ann Arbor stage	–	–	0.094	–	–	0.069
B symptoms	–	–	0.344	–	–	0.478
LDH level	–	–	0.459	–	–	0.348
Ki-67	–	–	0.902	–		0.478

Abbreviations: PFS, progression-free survival; OS, overall survival; HR, hazard ratio; CI, confidence interval; MTV, metabolic tumor volume; TLG, total lesion glycolysis; NCCN-IPI, National Comprehensive Cancer Network-International Prognostic Index; LDH, lactate dehydrogenase.

a MTV and TLG were dichotomized by cutoff values from ROC analysis.

b Cox-regression model

## DISCUSSION

^18^F-FDG PET/CT scan has been widely used in the management of DLBCL and there is growing evidence of the prognostic value of PET/CT parameters. SUVmax is the most commonly studied partly because of the convenience and high reproducibility of measurement. It reflects the metabolic activity of the most aggressive tumor cell [26]. However, MTV and TLG can provide more information than SUVmax and increasing number of evidences have indicated their potential value. More recently, a few researches have demonstrated the prognostic value of volume-based parameters in some tumors, such as malignant pleural mesothelioma, small lung cell cancer, etc [22–25]. Some retrospective studies also confirmed the prognostic functions of MTV or TLG in DLBCL patients [10–15, 18–21]. Mikhaeel even indicated MTV or TLG combined with early response in interim PET/CT could improve predictive value of DLBCL [21]. Specially, in the IELSG 26 study conducted by Ceriani and colleagues, 125 patients with primary mediastinal large B-cell lymphoma were prospectively enrolled and statistics revealed TLG was the only predictive factor of PFS and OS in multivariate analysis [14].

In our study, we have demonstrated that, both MTV and TLG have the potential to predict PFS and OS in DLBCL patients treated with R-CHOP therapy. TLG is the only independent factor for predicting survivals and a high TLG value is significantly associated with poor outcomes in DLBCL. MTV, NCCN-IPI and Ann Arbor stage failed to predict survivals independently. Our conclusion is nearly consistent with four retrospective studies respectively conducted by Esfahani, Kim, Ceriani and Xie, in despite of different patient population and different statistical methods [11, 13, 14, 19]. Song and colleagues found MTV as an independent factor of outcome in patients with primary gastrointestinal DLBCL and DLBCL with bone marrow involvement [10, 18]. Another study conducted by the same group indicated that TMV had more potential power than Ann Arbor stage in the DLBCL patients of stage II/III without extranodal site involvement [15]. Although we have found the association between MTV and survivals in univariate analysis, the multivariate analysis indicates TLG is the only independent predictive factor. TLG was not involved into the multivariate analysis in the above three researches, which mainly causes the discordant results with ours. Sasanelli et al found MTV as an independent factor of outcome in patients with DLBCL while TLG failed to be independently predictive [12]. The discordance may be caused by different therapies. Cottereau et al found MTV combined with molecular characteristics including GCB, MYC and BCL-2 could improve classification of DLBCL patients with poor prognosis [20]. However, in our study, no difference was found in PFS and OS between GCB and non-GCB groups. Two retrospective studies stated conclusions opposite to ours [16, 17]. Gallicchio et al found the SUVmax rather than MTV and TLG remained the only predictor for PFS in DLBCL patients and the data even showed poor outcome with lower values of SUVmax [16]. We hold the view that the statistical methods had drawbacks as multivariate analysis was not included. The other research conducted by Adams et al argued that SUVmax, MTV and TLG do not provide any prognostic information in DLBCL beyond which can already be obtained by NCCN-IPI [17]. In our study, statistics indicated association between NCCN-IPI and survivals, but TLG was more powerful in predictive ability.

Statistics in this study also revealed that the patients with higher TLG or MTV have more risk of relapse or disease progression, even though they got remission in R-CHOP therapy, as is shown in Figure 2 and Figure 3. In our opinion, this result is meaningful in clinical practice. For those patients with high TLG level, we should pay more attention to the treatment strategies and follow-up.

In DLBCL, TLG of baseline PET is the only quantitative parameter which accurately reflects tumor burden. But difference in measuring methods restricts its use. We find an interesting phenomenon that the cutoff value of dichotomizing MTV and TLG in our study and the previous studies differs in wide disparity. It partly results from different marginal threshold to calculate MTV. To the best of our knowledge, there are no published technical references about methodology of measuring MTV. According to previous studies in DLBCL, two methods are commonly used to decide the marginal threshold [10–18, 20, 21]. One is the proportion of SUVmax in one lesion, ranging from 25% to 50%. We consider this method disadvantageous. Using the proportion of SUVmax as the threshold may not be able to estimate metabolic volume correctly because SUVmax differs in different lesions of DLBCL. When SUVmax is a relatively high value, we may underestimate the volume. For instance, when a threshold of 41% of SUVmax is used to measure the volume of a mass with SUVmax of 20, it means the hyper-metabolic lesion with SUV less than 8.2 was not included in the region of interest (ROI). So the ideal proportion may change according to SUVmax, and that could be the main reason of the inconsistent proportion in previous studies. The other is an absolute cutoff value of SUV and 2.5 is commonly used, as suggested by Freudenberg et al [26]. SUV can be affected by various factors including different PET scans, a poor intravenous injection, time after injection or variable uptake time, so SUV of 2.5 is not an ideal marginal threshold for our study. Only one study used liver SUVmean plus 2SDs as a marginal threshold [18]. We used a method with a threshold equal to 3SDs above normal liver mean SUV determined in a standard-sized ROI of 3cm in diameter. Our method was similar to PERCIST, in which mean SUL (SUV lean) in normal liver plus 3SDs is recommended [27]. This per-patient adapted threshold based on liver background is able to reduce the effects of different PET systems and other technical and patient-dependent factors in our study. A previous research conducted by Kanoun et al evaluated the influence of methodology of calculating MTV in Hodgkin's lymphoma. MTV values were proven to be significantly influenced by methodology, but MTV values were predictive for PFS in all methodologies [28]. We consider that the conclusion may also apply to DLBCL. It is necessary to normalize the measuring method if we need to apply an accurate cutoff value of TLG to the management of DLBCL.

## CONCLUSION

Our study indicates that the baseline TLG is an independent predictor for survivals in DLBCL patients treated with R-CHOP. High TLG level is associated with poor PFS and OS. Baseline TLG will help clinicians to identify the risk subgroups and make adjustments to the treatment strategies in DLBCL. Future efforts should be made to standardize the methodology of measuring MTV and TLG, and to confirm prognostic value of TLG in more prospective multicenter studies.

## MATERIALS AND METHODS

### Patient population

We performed a retrospective analysis of 91 patients (44 men and 47 women; age range, 17-83y) with newly diagnosed DLBCL. All patients had undergone ^18^F-FDG PET/CT scans before appropriate treatment at Shanghai Jiaotong University-Affiliated Ren Ji Hospital between March 2005 and October 2014. Inclusion criteria for this study were as follows: (a) the diagnose of DLBCL was pathologically confirmed; (b) first-line therapy with 6 or 8 cycles of R-CHOP therapy; (c) no history of other malignant tumors; (d) no central nervous system involvement of DLBCL; (e) complete clinical and pathological information was available. This study was approved by Institutional Review Board of Ren Ji Hospital.

### ^18^F-FDG PET/CT imaging procedures

Baseline PET/CT images were acquired by dedicated PET/CT scanners (2005-2010, GE discovery ST 8-slice CT in PET/CT; after 2010, Siemens Biograph 64 rows of PET/CT) on all patients within 14 days prior to chemotherapy. All patients received an intravenous injection of ^18^F- FDG (3.7 MBq/kg, or 0.1mCi/kg) after fasting for at least 6 hours. Blood glucose was also measured before the injection to make sure it was no more than 140 mg/dL. The mean uptake time was 50±6 minutes. CT scans were acquired 120 kV and 140 mA (mean), with a section width of 5.0 mm. PET images were reconstructed iteratively with CT data for attenuation correction.

### ^18^F-FDG PET parameters

The ^18^F-FDG PET images were analyzed by two experienced independent observers blinded from any clinical information. On a dedicated workstation, Philips IntelliSpace Portal 7.0 (Philips, Amsterdam, Holland), metabolic parameters were measured in all baseline PET/CT scans. SUVmax was calculated automatically by the workstation. MTV was measured by setting the tumor marginal threshold of liver SUVmean plus 3SDs. SUVmean in liver was calculated in a standard-sized ROI of 3cm in diameter [27]. TLG was the sum of the products of MTV and SUVmean in all measured lesions.

### Statistical methods

MTV and TLG were dichotomized by respective median values and differences in clinical and pathological factors between groups were analyzed by Pearson's chi-square test. The PET metabolic parameters were analyzed using receiver-operating characteristic (ROC) curve to estimate the optimal cutoff values. Overall survival was defined as the time from diagnosis to death or the last follow-up visit, and progression-free survival was from initial treatment to disease progression, death or last follow-up visit. Survival curves were derived by the Kaplan-Meier method in two groups dichotomized by optimal cutoff values of PET parameters and the between-group difference was evaluated by Log-rank test. Cox-regression model was used to identify independently predictive variables from clinical, pathologic and imaging variables. The hazard ratio (HR) and its 95%CI were also calculated by Cox-regression model. A p value less than 0.05 was considered to be statistically significant. Statistical analysis was conducted using SPSS, version 20.0 (IBM corporation, NY, USA).

## References

[1] Flowers CR, Sinha R, Vose JM (2010). Improving outcomes for patients with diffuse large B-cell lymphoma. CA Cancer J Clin.

[2] Friedberg JW (2011). Relapsed/refractory diffuse large B-cell lymphoma. Hematology Am Soc Hematol Educ Program 2011.

[3] Hutchings M, Barrington SF (2009). PET/CT for therapy response assessment in lymphoma. J Nucl Med.

[4] (1993). A predictive model for aggressive non-Hodgkin's lymphoma. The International Non-Hodgkin's Lymphoma Prognostic Factors Project. N Engl J Med.

[5] Bari A, Marcheselli L, Sacchi S, Marcheselli R, Pozzi S, Ferri P, Balleari E, Musto P, Neri S, Aloe Spiriti MA, Cox MC (2010). Prognostic models for diffuse large B-cell lymphoma in the rituximab era: a never-ending story. Ann Oncol.

[6] Zhou Z, Sehn LH, Rademaker AW, Gordon LI, Lacasce AS, Crosby-Thompson A, Vanderplas A, Zelenetz AD, Abel GA, Rodriguez MA, Nademanee A, Kaminski MS, Czuczman MS (2014). An enhanced International Prognostic Index (NCCN-IPI) for patients with diffuse large B-cell lymphoma treated in the rituximab era. Blood.

[7] Juweid ME, Stroobants S, Hoekstra OS (2007). Use of positron emission tomography for response assessment of lymphoma: consensus of the imaging subcommittee of international harmonization project in lymphoma. J Clin Oncol.

[8] Baba S, Abe K, Isoda T, Maruoka Y, Sasaki M, Honda H (2011). Impact of FDG-PET/CT in the management of lymphoma. Ann Nucl Med.

[9] Cheson BD, Pfistner B, Juweid ME, Gascoyne RD, Specht L, Horning SJ, Coiffier B, Fisher RI, Hagenbeek A, Zucca E, Rosen ST, Stroobants S, Lister TA (2007). International Harmonization Project on Lymphoma. Revised response criteria for malignant lymphoma. J Clin Oncol.

[10] Song MK, Chung JS, Shin HJ, Moon JH, Lee JO, Lee HS, Lee SM, Lee GW, Lee SE, Kim SJ (2012). Prognostic value of metabolic tumor volume on PET/CT in primary gastrointestinal diffuse large B cell lymphoma. Cancer Sci.

[11] Esfahani SA, Heidari P, Halpern EF, Hochberg EP, Palmer EL, Mahmood U (2013). Baseline total lesion glycolysis measured with (18)F-FDG PET/CT as a predictor of progression-free survival in diffuse large B-cell lymphoma: a pilot study. Am J Nucl Med Mol Imaging.

[12] Sasanelli M, Meignan M, Haioun C, Berriolo-Riedinger A, Casasnovas RO, Biggi A, Gallamini A, Siegel BA, Cashen AF, Véra P, Tilly H, Versari A, Itti E (2014). Pretherapy metabolic tumour volume is an independent predictor of outcome in patients with diffuse large B-cell lymphoma. Eur J Nucl Med Mol Imaging.

[13] Kim TM, Paeng JC, Chun IK, Keam B, Jeon YK, Lee SH, Kim DW, Lee DS, Kim CW, Chung JK, Kim IH, Heo DS (2013). Total lesion glycolysis in positron emission tomography is a better predictor of outcome than the International Prognostic Index for patients with diffuse large B cell lymphoma. Cancer.

[14] Ceriani L, Martelli M, Zinzani PL, Ferreri AJ, Botto B, Stelitano C, Gotti M, Cabras MG, Rigacci L, Gargantini L, Merli F, Pinotti G, Mannina D (2015). Utility of baseline 18FDG-PET/CT functional parameters in defining prognosis of primary mediastinal (thymic) large B-cell lymphoma. Blood.

[15] Song MK, Chung JS, Shin HJ, Lee SM, Lee SE, Lee HS, Lee GW, Kim SJ, Lee SM, Chung DS (2012). Clinical significance of metabolic tumor volume by PET/CT in stages II and III of diffuse large B cell lymphoma without extranodal site involvement. Ann Hematol.

[16] Gallicchio R, Mansueto G, Simeon V, Nardelli A, Guariglia R, Capacchione D, Soscia E, Pedicini P, Gattozzi D, Musto P, Storto G (2014). F-18 FDG PET/CT quantization parameters as predictors of outcome in patients with diffuse large B-cell lymphoma. Eur J Haematol.

[17] Adams HJ, de Klerk JM, Fijnheer R, Heggelman BG, Dubois SV, Nievelstein RA, Kwee TC (2015). Prognostic superiority of the National Comprehensive Cancer Network International Prognostic Index over pretreatment whole-body volumetric-metabolic FDG-PET/CT metrics in diffuse large B-cell lymphoma. Eur J Haematol.

[18] Song MK, Yang DH, Lee GW, Lim SN, Shin S, Pak KJ, Kwon SY, Shim HK, Choi BH, Kim IS, Shin DH, Kim SG, Oh SY (2016). High total metabolic tumor volume in PET/CT predicts worse prognosis in diffuse large B cell lymphoma patients with bone marrow involvement in rituximab era. Leuk Res.

[19] Xie M, Zhai W, Cheng S, Zhang H, Xie Y, He W (2016). Predictive value of F-18 FDG PET/CT quantization parameters for progression-free survival in patients with diffuse large B-cell lymphoma. Hematology.

[20] Cottereau AS, Lanic H, Mareschal S, Meignan M, Vera P, Tilly H, Jardin F, Becker S (2016). Molecular profile and FDG-PET/CT total metabolic tumor volume improve risk classification at diagnosis for patients with diffuse large B-cell lymphoma. Clin Cancer Res.

[21] Mikhaeel NG, Smith D, Dunn JT, Phillips M, Møller H, Fields PA, Wrench D, Barrington SF (2016). Combination of baseline metabolic tumor volume and early response on PET/CT improves progression-free survival prediction in DLBCL. Eur J Nucl Med Mol Imaging.

[22] Lee HY, Hyun SH, Lee KS, Kim BT, Kim J, Shim YM, Ahn MJ, Kim TS, Yi CA, Chung MJ (2010). Volume-based parameter of 18F-FDG PET/CT in malignant pleural mesothelioma: prediction of therapeutic response and prognostic implications. Ann Surg Oncol.

[23] Arslan N, Tuncel M, Kuzhan O, Alagoz E, Budakoglu B, Ozet A, Ozguven MA (2011). Evaluation of outcome prediction and disease extension by quantitative 2-deoxy-2-[18F] fluoro-D-glucose with positron emission tomography in patients with small cell lung cancer. Ann Nucl Med.

[24] Costelloe CM, Macapinlac HA, Madewell JE, Fitzgerald NE, Mawlawi OR, Rohren EM, Raymond AK, Lewis VO, Anderson PM, Bassett RL, Harrell RK, Marom EM (2009). 18F-FDG PET/CT as an indicator of progression-free and overall survival in osteosarcoma. J Nucl Med.

[25] Fonti R, Larobina M, Del Vecchio S, De Luca S, Fabbricini R, Catalano L, Pane F, Salvatore M, Pace L (2012). Metabolic tumor volume assessed by 18F-FDG PET/CT for the prediction of outcome in patients with multiple myeloma. J Nucl Med.

[26] Freudenberg LS, Antoch G, Schütt P, Beyer T, Jentzen W, Müller SP, Görges R, Nowrousian MR, Bockisch A, Debatin JF (2004). FDG-PET/CT in re-staging of patients with lymphoma. Eur J Nucl Med Mol Imaging.

[27] Wahl RL, Jacene H, Kasamon Y, Lodge MA (2009). From RECIST to PERCIST: Evolving Considerations for PET response criteria in solid tumors. J Nucl Med.

[28] Kanoun S, Tal I, Berriolo-Riedinger A, Rossi C, Riedinger JM, Vrigneaud JM, Legrand L, Humbert O, Casasnovas O, Brunotte F, Cochet A (2015). Influence of software tool and methodological aspects of total metabolic tumor volume calculation in baseline [18F] FDG PET to predict survival in Hodgkin lymphoma. PLoS One.

